# Cold-inducible RNA-binding protein might determine the severity and the presences of major/minor criteria for severe community-acquired pneumonia and best predicted mortality

**DOI:** 10.1186/s12931-020-01457-2

**Published:** 2020-07-20

**Authors:** Qi Guo, Wei-dong Song, Hai-yan Li, Ming Li, Xiao-ke Chen, Hui Liu, Hong-lin Peng, Hai-qiong Yu, Nian Liu, Zhong-dong Lü, Li-hua Liang, Qing-zhou Zhao, Mei Jiang

**Affiliations:** 1 Department of Pulmonary and Critical Care Medicine, Shenzhen Hospital, Peking University, Lianhua road No. 1120, Shenzhen, 518036 Guangdong China; 2 Department of Pulmonary and Critical Care Medicine, The Eighth Affiliated Hospital (Shenzhen Futian), Sun Yat-sen University, Shenzhen, 518033 Guangdong China; 3 Medical Department, The Eighth Affiliated Hospital (Shenzhen Futian), Sun Yat-sen University, Shenzhen, 518033 Guangdong China; 4 Department of Radiology, The Eighth Affiliated Hospital (Shenzhen Futian), Sun Yat-sen University, Shenzhen, 518033 Guangdong China; 5 State Key Laboratory of Respiratory Disease, National Clinical Research Center for Respiratory Disease, Guangzhou Institute of Respiratory Health, The First Affiliated Hospital, Guangzhou Medical University, Guangzhou, 510120 Guangdong China

**Keywords:** Community-acquired pneumonia, Biomarkers, Cold-inducible RNA-binding protein, Major/minor criteria, Severity, Mortality

## Abstract

**Background:**

Severity of community-acquired pneumonia (CAP) depends on microbial pathogenicity, load and virulence, and immune responses. The Infectious Disease Society of America and the American Thoracic Society (IDSA/ATS) minor criteria responsible for clinical triage of patients with CAP are of unequal weight in predicting mortality. It is unclear whether the IDSA/ATS major/minor criteria might be strongly and positively associated with the immune responses. It is warranted to explore this intriguing hypothesis.

**Methods:**

A prospective cohort study of 404 CAP patients was performed. Cold-inducible RNA-binding protein (CIRP) levels were measured using a sandwich-based enzyme-linked immunosorbent assay. The receiver operating characteristic curves were created and the areas under the curves were calculated to illustrate and compare the accuracy of the indices.

**Results:**

Severe CAP patients meeting the major criteria had the highest plasma concentrations of CIRP. The more the number of most predictive minor criteria strongly associated to mortality, i.e. arterial oxygen pressure/fraction inspired oxygen ≤ 250 mmHg, confusion, and uremia, present, the higher the CIRP level. Interestingly, the patients with non-severe CAP meeting the most predictive minor criteria demonstrated unexpectedly higher CIRP level compared with the patients with severe CAP not fulfilling the criteria. Procalcitonin (PCT), interleukin-6 (IL-6), C-reactive protein (CRP), sequential organ failure assessment (SOFA) and pneumonia severity index (PSI) scores, and mortality confirmed similar intriguing patterns. CIRP was strongly linked to PCT, IL-6, CRP, minor criteria, SOFA and PSI scores, and mortality (increased odds ratio 3.433). The pattern of sensitivity, specificity, positive predictive value, and Youden’s index of CIRP ≥ 3.50 ng/mL for predicting mortality was the optimal. The area under the receiver operating characteristic curve of CIRP was the highest among the indices.

**Conclusions:**

CIRP levels were strongly correlated with the IDSA/ATS major/minor criteria. CIRP might determine the severity and the presences of major/minor criteria and best predicted mortality, and a CIRP of ≥ 3.50 ng/mL might be more valuable cut-off value for severe CAP, suggesting that CIRP might be a novel and intriguing biomarker for pneumonia to monitor host response and predict mortality, which might have implications for more accurate clinical triage decisions.

## Background

The mortality of community-acquired pneumonia (CAP) remains unacceptably high, in spite of substantial advances in treatment and the emergence of well validated pneumonia severity scoring systems [1–5]. Therefore, the assessment of severity is still crucial in the management of CAP. In 2007, the Infectious Disease Society of America and the American Thoracic Society (IDSA/ATS) issued guidelines, with the aim to guide intensive care unit (ICU) admission, which defined severe CAP—when one of two major criteria or three of nine minor criteria are fulfilled [2]. The minor criteria established by IDSA/ATS are of unequal weight in predicting mortality, and arterial oxygen pressure/fraction inspired oxygen (PaO_2_/FiO_2_) ≤ 250 mmHg, confusion, and uremia were the most predictive minor criteria strongly associated to mortality [6–9]. Interestingly, we further found that the combination of the most predictive minor criteria predicted more severity and higher mortality in patients with CAP compared with others [10], and that the patients with non-severe CAP fulfilling the most predictive minor criteria demonstrated unexpectedly higher sequential organ failure assessment (SOFA) and pneumonia severity index (PSI) scores and mortality rates compared with the patients with severe CAP not meeting the most predictive minor criteria [11]. Salih et al. [12] reported that the minor criteria could be simplified by removing three infrequent variables, but could not improve the prediction of mortality and ICU admission. We further discovered that the minor criteria could be simplified to five variables and then be modified to orchestrate improvements in predicting mortality in CAP patients [13]. Hence, the IDSA/ATS major/minor criteria might at least be stratified to predict better (Table 1). On the basis of the limitations of IDSA/ATS minor criteria, including cumbersome application and less accurate mortality prediction, additional more accurate biomarkers are necessary to improve our ability to predict bad CAP outcomes and then aid clinical triage decisions.

**Table 1:** Stratified IDSA/ATS major/minor criteria for severe CAP

Characteristic	
Major criteria	
Invasive mechanical ventilation	
Septic shock with the need for vasopressors	
Minor criteria	
High risk categories (the most predictive minor criteria)	PaO_2_/FiO_2_ ≤ 250 mmHg
	Confusion/disorientation
	Uremia (BUN level, ≥ 20 mg/dL)
Low risk categories	Respiratory rate ≥ 30 breaths/min
	Multilobar infiltrates
	Leukopenia (WBC count, < 4000 cells/mm3)
	Thrombocytopenia (platelet count, < 100,000 cells/mm3)
	Hypothermia (core temperature, < 36 °C)
	Hypotension requiring aggressive fluid resuscitation

Stratified IDSA/ATS major/minor criteria defined severe CAP as what the original criteria did, but the patients fulfilling the high risk categories might present more severity and higher mortality compared with those meeting the low risk categories. On the basis of the presences of major criteria and most predictive minor criteria, severe CAP patients consisted of those meeting one or two of the major criteria, three of the most predictive minor criteria, two of the most predictive minor criteria, one of the most predictive minor criteria and none of the most predictive minor criteria, respectively, and non-severe CAP patients incorporated those fulfilling two of the most predictive minor criteria, one of the most predictive minor criteria and none of the most predictive minor criteria, respectively

*IDSA/ATS* The Infectious Disease Society of America and the American Thoracic Society, *CAP* community-acquired pneumonia, *PaO*_*2*_*/FiO*_*2*_ Arterial oxygen pressure/fraction inspired oxygen, *BUN* Blood urine nitrogen, *WBC* White blood cell

Severity of CAP depends on microbial pathogenicity, load and virulence, and immune responses to infection. Both exogenous pathogen-associated molecular pattern molecules and endogenous damaged-associated molecular pattern molecules are recognized by immune cells through a group of pattern-recognition receptors, e.g. Toll-like receptors [14–16]. After engaging with the receptors responsible for the perception of signature molecules that herald infection, signal transduction is activated, triggering inflammatory responses, e.g. the release of numerous inflammatory mediators including cytokines, chemokines, and vasoactive peptides [17–19]. Sepsis is a systemic response to infection, and symptoms are produced by host defense systems rather than by the invading pathogens. Exaggerated immune responses are usually shown in patients with CAP, which may lead to severe sepsis and multiple organ failure. Cold-inducible RNA-binding protein (CIRP) is a damage-associated molecular pattern molecule that plays a pivotal role in triggering inflammatory response, and antisera to CIRP attenuate shock-induced inflammation, tissue injury, and lethality [20]. CIRP induces excessive neutrophil extracellular traps, which cause inflammation and tissue damage, in the lungs during sepsis [21]. Intravenous injection of recombinant murine CIRP in C57BL/6 mice causes lung injury, evidenced by vascular leakage, edema, increased leukocyte infiltration and cytokine production in the lung tissue. The CIRP-induced lung damage is accompanied with endothelial cell activation and pyroptosis [22]. Therefore, we speculated that the expression of CIRP triggered by invading microorganisms might determine disease severity and mortality in patients with sepsis. An elevated plasma concentration of CIRP was significantly associated with poor prognosis among patients with sepsis [23]. It is unclear whether the major/minor criteria might be strongly and positively associated with the immune responses, e.g. CIRP. Therefore, a prospective cohort study was conducted to determine the intriguing hypothesis.

## Materials and methods

### Design and setting

We performed a prospective two-centre cohort study of 404 adult patients with CAP among 1611 eligible patients between 2015 and 2018 at the Departments of Pulmonary and Critical Care Medicine in two Chinese affiliated tertiary hospitals of two medical universities. The two departments all consisted of general ward and respiratory ICU.

### Criteria for enrollment

CAP was defined as an acute infection of the pulmonary parenchyma associated with an acute infiltrate on the chest radiograph with two or more symptoms including fever (> 38 °C), hypothermia (< 36 °C), rigors, sweats, new cough or change in color of respiratory secretions, chest discomfort or dyspnoea [9]. Patients who were younger than 18 years, who had been hospitalized during the 28 days preceding the study, who had severe immunosuppression, active tuberculosis, or end-stage diseases, who had a written “do not resuscitate” order, or whose baseline status was unconscious before suffering from pneumonia, were excluded.

### Clinical management

The study was conducted in accordance with the principles described in human experimentation guidelines of the United States Department of Health and Human Services. Patients with CAP were admitted and attended by respiratory physicians in accordance with the IDSA/ATS guidelines [2] and the Surviving Sepsis Campaign guidelines [24, 25]. The empirical antibiotic regimens were adherence to the guidelines, and then adjusted based on subsequently cultured pathogens. All patients were discharged home when they reached clinical stability and became afebrile.

### Grouping participants

Stratified IDSA/ATS major/minor criteria defined severe CAP as what the original criteria did (Table 1). One thousand six hundred thirty-seven consecutive patients with CAP were assessed and 26 cases were excluded from the cohort due to exclusion criteria. Patients were enrolled in order until the target number was reached for each group. Therefore, 404 discontinuous patients among 1611 eligible patients were enrolled and then assigned to 8 groups, i.e. severe CAP patients meeting one or two of the major criteria, three of the most predictive minor criteria (no any major criteria, similarly hereinafter), two of the most predictive minor criteria, one of the most predictive minor criteria and none of the most predictive minor criteria, respectively, and non-severe CAP patients fulfilling two of the most predictive minor criteria, one of the most predictive minor criteria and none of the most predictive minor criteria, respectively. Sixty patients would be included in each group expectedly, but unfortunately there were not enough severe CAP patients meeting the major criteria or three of the most predictive minor criteria (only 16 and 28 patients, respectively). In the other groups, only the first 60 patients fulfilling the corresponding criteria were enrolled.

### Sample collection

Plasma specimens were obtained from the patients with CAP on admission. Peripheral blood was collected into EDTA tubes and centrifuged at 3000 g at 4 °C for 10 min, and then the isolated plasma was frozen at − 80 °C.

### Outcome

The main outcome measures were the plasma concentration of CIRP on admission and 28-day mortality. Secondary outcomes incorporated SOFA and PSI scores, and the concentrations of procalcitonin (PCT), interleukin-6 (IL-6) and C-reactive protein (CRP) in sera on admission.

### Data collection

All the patients had chest radiographys and/or computer tomography (CT) scans. The frontal and lateral chest radiographic findings and CT scan images were classified independently by two senior radiologists (LH Liang and QZ Zhao). Clinical and diagnostic data, and radiological features were collected. The plasma concentrations of CIRP were measured in duplicate using a sandwich-based enzyme-linked immunosorbent assay (ELISA; CUSABIO, Wuhan, China). SOFA and PSI scores on admission were calculated. Laboratory variables including PCT, IL-6 and CRP were measured by the hospital clinical laboratories. The statistician was blinded to the study.

### Statistical analysis

All statistical analyses were performed with Statistical Package for the Social Science for Windows version 16.0 (SPSS, Chicago, IL, USA) and MedCalc version 19.1.3 (Mariakerke, Belgium). Categorical variables and continuous variables were reported as the percentages and the mean ± standard deviation (SD), respectively. Chi-Square test, one-way ANOVA, univariate logistic regression, and Spearman rank correlation were employed. The receiver operating characteristic (ROC) curves were created and the areas under the curves (AUCs) were calculated to illustrate and compare the accuracy of the indices. The sensitivities, specificities, positive predictive values (PPVs), negative predictive values (NPVs), and Youden’s indices were also calculated. A *p* value of < 0.05 was considered statistically significant.

## Results

### Baseline characteristics of study cohort

Baseline characteristics of the patients were shown in Table 2. Sputum was collected into a sterile container and cultured according to standard procedures, but the pathogens were detected in a minority of the patients. Therefore, the data were not shown.

**Table 2:** Baseline characteristics of study cohort (Mean ± SD, *n* = 404)

Characteristic	Result
Age (yrs)	65.14 ± 19.60
Male sex (%)	38.9
Hospital Length of stay (days)	12.6 ± 9.8
Age ≥ 65 yrs. (%) (No.)	57.4 (232)
Comorbidities (%) (No.)	
Hypertension	41.9 (169)
Coronary heart disease	19.3 (78)
Heart failure	5.2 (21)
Chronic obstructive pulmonary disease	8.4 (34)
Diabetes mellitus	11.5 (46)
Chronic renal insufficiency	6.7 (27)
Liver disease	6.1 (25)
Nervous system disease	7.8 (32)
Tumour	10.9 (44)
Alcohol abuse (%) (No.)	2.9 (12)
Smoking (%) (No.)	31.4 (127)
Respiratory rate ≥ 30 breaths/min (%) (No.)	26.5 (107)
PaO_2_/FiO_2_ ≤ 250 mmHg (%) (No.)	32.7 (132)
Multilobar infiltrates (%) (No.)	67.1 (271)
Confusion (%) (No.)	13.1 (53)
Uremia (%) (No.)	38.6 (156)
Leukopenia (%) (No.)	14.6 (59)
Thrombocytopenia (%) (No.)	9.2 (37)
Hypothermia (%) (No.)	12.4 (50)
Hypotension (%) (No.)	35.6 (144)

*PaO*_*2*_*/FiO*_*2*_ Arterial oxygen pressure/fraction inspired oxygen

### CIRP, PCT, IL-6, CRP, SOFA and PSI scores, and mortality according to the major/minor criteria present

Severe CAP patients meeting the major criteria had the highest plasma concentrations of CIRP (Table 3). Among the severe CAP patients meeting the minor criteria, the number of IDSA/ATS minor criteria strongly associated to mortality (i.e. PaO_2_/FiO_2_ ≤ 250 mmHg, confusion and uremia) was directly correlated with CIRP level. Interestingly, the patients with non-severe CAP meeting the most predictive minor criteria demonstrated unexpectedly higher CIRP level compared with the patients with severe CAP not fulfilling the criteria. Among the non-severe CAP patients, the numbers of most predictive minor criteria present were also strongly and positively associated with the CIRP levels. All the differences between the groups were significant (*p* <  0.001), except for that between severe CAP patients meeting one of the most predictive minor criteria and non-severe CAP patients fulfilling two of the most predictive minor criteria. PCT, IL-6, CRP, SOFA and PSI scores, and mortality confirmed similar and intriguing paradigms (Table 3).

**Table 3:** CIRP, PCT, IL-6, CRP, SOFA and PSI scores, and mortality according to the IDSA/ATS major/minor criteria present (Mean ± SD, *n* = 404)

CAP patients	CIRP (ng/mL)	PCT (ng/mL)	IL-6 (pg/mL)	CRP (mg/L)	SOFA score	PSI score	Death (%)
Severe CAP patients meeting the major criteria (*n* = 16)	5.82 ± 0.75	2.90 ± 0.49	181.45 ± 15.01	135.52 ± 13.78	6.88 ± 1.41	153.69 ± 10.74	8 (50)
Severe CAP patients meeting three of the most predictive minor criteria (*n* = 28)	4.51 ± 0.49	2.33 ± 0.55	163.00 ± 16.36	117.25 ± 15.77	5.86 ± 1.38	134.57 ± 5.65	8 (28.6)
Severe CAP patients meeting two of the most predictive minor criteria (*n* = 60)	3.41 ± 0.27	1.82 ± 0.47	140.37 ± 11.47	82.93 ± 11.80	3.90 ± 1.49	125.50 ± 6.05	10 (16.7)
Severe CAP patients meeting one of the most predictive minor criteria (*n* = 60)	2.78 ± 0.31	0.96 ± 0.29	130.12 ± 10.20	71.69 ± 11.33	3.23 ± 1.51	117.22 ± 6.87	7 (11.7)
Severe CAP patients meeting none of the most predictive minor criteria (*n* = 60)	1.67 ± 0.11	0.31 ± 0.09	67.08 ± 5.97	23.65 ± 5.75	1.53 ± 1.50	65.60 ± 4.79	2 (3.3)
Non-severe CAP patients meeting two of the most predictive minor criteria (*n* = 60)	2.85 ± 0.31	0.74 ± 0.36	121.61 ± 8.25	51.44 ± 12.50	3.48 ± 1.55	108.40 ± 5.34	5 (8.3)
Non-severe CAP patients meeting one of the most predictive minor criteria (*n* = 60)	2.27 ± 0.50	0.53 ± 0.11	108.84 ± 7.04	37.60 ± 10.92	3.03 ± 1.55	74.02 ± 6.57	4 (6.7)
Non-severe CAP patients meeting none of the most predictive minor criteria (*n* = 60)	0.92 ± 0.13	0.20 ± 0.08	36.45 ± 16.08	11.45 ± 5.48	0.52 ± 0.79	36.75 ± 2.38	0 (0)
*F* or *x*^*2*^ value	643.360	333.307	790.423	586.484	75.037	2003.649	48.707
*p* value	< 0.001	< 0.001	< 0.001	< 0.001	< 0.001	< 0.001	< 0.001
Rank correlation coefficient (*r*_s_) value	0.786	0.806	0.811	0.836	0.579	0.858	0.275
*p* value	< 0.001	< 0.001	< 0.001	< 0.001	< 0.001	< 0.001	< 0.001

*CIRP* Cold-inducible RNA-binding protein, *PCT* Procalcitonin, *IL-6* Interleukin-6, *CRP* C-reactive protein, *SOFA* Sequential organ failure assessmentm, *PSI* Pneumonia severity index, *IDSA/ATS* The Infectious Disease Society of America and the American Thoracic Society, *CAP* community-acquired pneumonia

### Relationships between CIRP and mortality, minor criteria, SOFA and PSI scores, PCT, IL-6 and CRP

CIRP demonstrated the strongest relationship with mortality [*x*^*2*^ = 323.972, *p* <  0.001. Odds ratio (OR), 3.433; 95% confidence interval (CI), 2.447–4.816; *p* <  0.001. Table 4]. The correlations of CIRP with minor criteria and SOFA and PSI scores were stronger than those of PCT, IL-6 and CRP, respectively, and CIRP was also strongly associated with PCT, IL-6 and CRP, indicating that CIRP might determine the severity of CAP and the presences of minor criteria and suggesting that CIRP might be a novel and intriguing biomarker for pneumonia to monitor host response.

**Table 4:** Relationships between CIRP and mortality, minor criteria, SOFA and PSI scores, PCT, IL-6 and CRP (*n* = 404)

*r*_s_	PCT	IL-6	CRP	minor criteria	SOFA score	PSI score	Mortality
CIRP	0.875	0.883	0.872	0.550	0.843	0.952	0.402
PCT		0.897	0.896	0.426	0.666	0.909	0.319
IL-6			0.907	0.325	0.673	0.920	0.287
CRP				0.367	0.665	0.917	0.289
minor criteria					0.390	0.661	0.184
SOFA score						0.803	0.377
PSI score							0.387

*CIRP* Cold-inducible RNA-binding protein, *SOFA* Sequential organ failure assessment, *PSI* Pneumonia severity index, *PCT* Procalcitonin, *IL-6* Interleukin-6, *CRP* C-reactive protein, *r*_s_: Rank correlation coefficient

All *p* values were less than 0.001

### Relationship between cut-off values and ranks of CIRP level and risk of mortality

Several cut-off values and ranks of CIRP level were chosen. The mortality rose sharply as the CIRP level increased, except for that in the rank of 1.75–2.25 ng/mL (Table 5).

**Table 5:** Relationship between cut-off values and ranks of CIRP level and risk of mortality (*n* = 404)

CIRP (ng/mL)	NO. (%) patients	NO. (%) deaths
Cut-off value		
≥ 0.75	404 (100)	44 (10.9)
≥ 1.75	291 (72.0)	42 (14.4)
≥ 2.25	247 (61.1)	42 (17.0)
≥ 2.75	196 (48.5)	41 (20.9)
≥ 3.50	67 (16.6)	27 (40.3)
≥ 4.50	27 (6.7)	16 (59.3)
Rank		
< 1.75	113 (28.0)	2 (1.8)
1.75–2.25	45 (11.1)	0 (0)
2.26–2.75	54 (13.4)	1 (1.9)
2.76–3.50	125 (30.9)	14 (11.2)
3.51–4.50	41 (10.1)	11 (26.8)
> 4.50	26 (6.4)	16 (61.5)

*CIRP* Cold-inducible RNA-binding protein

### Accuracy of mortality prediction

Table 6 described the sensitivities, specificities, and predictive values of cut-off values of CIRP level for prediction of mortality. The pattern of sensitivity, specificity, PPV, and Youden’s index of the plasma concentration of CIRP ≥ 3.50 ng/mL for predicting mortality was the optimal. Therefore, the plasma concentration of CIRP ≥ 3.50 ng/mL might be more valuable cut-off value for severe CAP.

**Table 6:** Test characteristics of cut-off values of CIRP for mortality in patients hospitalized with CAP (*n* = 404)

CIRP (ng/mL)	Sensitivity (%)	Specificity (%)	PPV (%)	NPV (%)	Youden’s index
≥ 0.75	100	0	10.9	0	0
≥ 1.75	95.5	30.8	14.4	98.2	0.26
≥ 2.25	95.5	43.1	17.0	98.7	0.39
≥ 2.75	93.2	56.9	20.9	98.6	0.50
≥ 3.50	61.4	88.9	40.3	95.0	0.50
≥ 4.50	36.4	96.9	59.3	92.6	0.33

*CIRP* Cold-inducible RNA-binding protein, *CAP* Community-acquired pneumonia, *PPV* Positive predictive value, *NPV* Negative predictive value

**Fig. 1:**
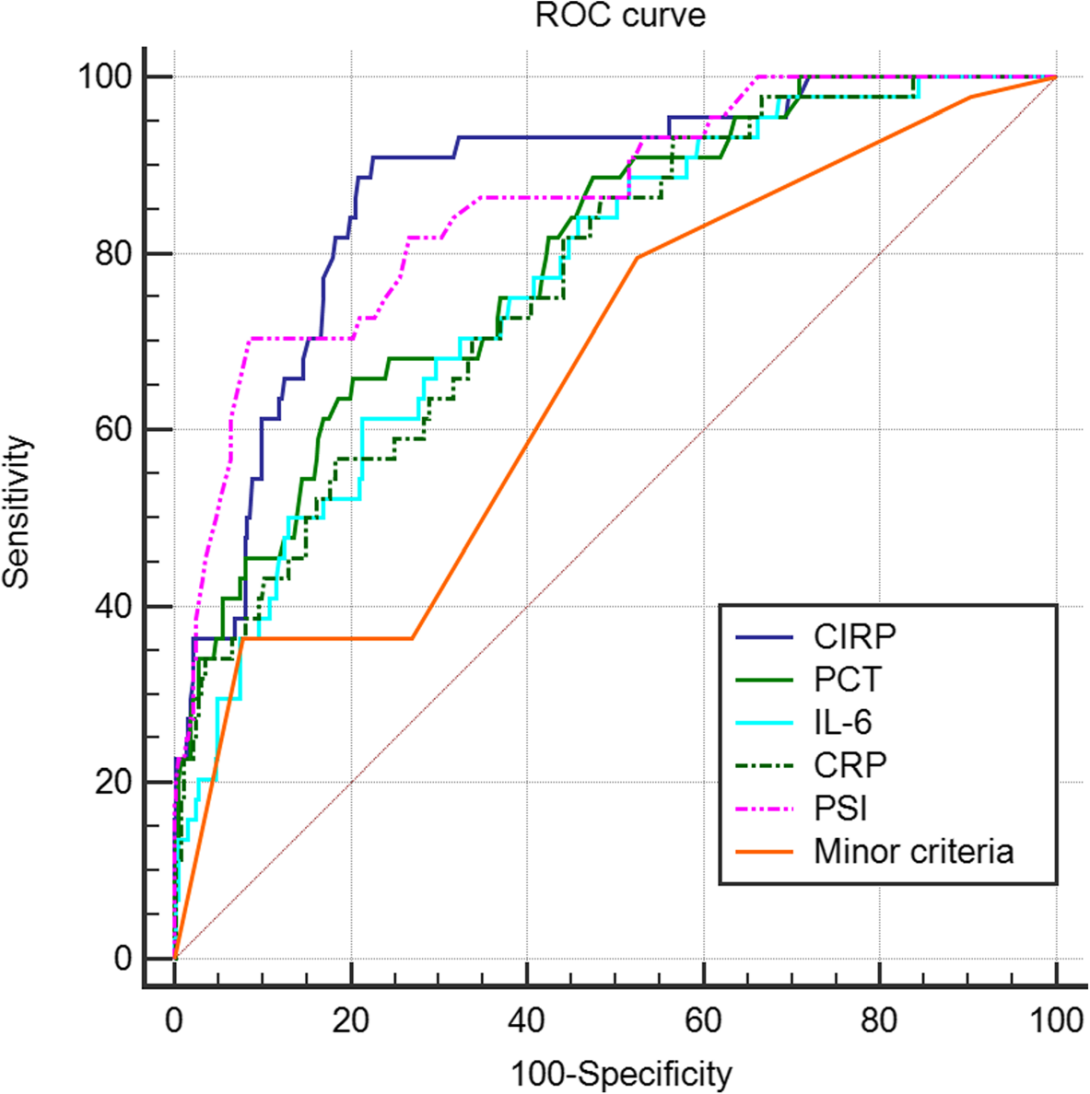
ROC curves for mortality prediction by CIRP, PCT, IL-6, CRP, PSI, and minor criteria. ROC: The receiver operating characteristic. CIRP: Cold-inducible RNA-binding protein. PCT: Procalcitonin. IL-6: Interleukin-6. CRP: C-reactive protein. PSI: Pneumonia severity index

The ROC curves for CIRP, PCT, IL-6, CRP, SOFA and PSI scores, and minor criteria in the study population illustrated the differences in accuracy of mortality prediction (Table 7 and Fig. 1). The higher accuracy was illustrated by the higher AUC value. Among the 7 indices, CIRP was performed best.

**Table 7:** AUC values for different rules to predict mortality and their comparisons

Feature	AUC value	Standard error	95% CI
CIRP	0.873	0.0269	0.836–0.904
PCT	0.795	0.0349	0.753–0.834
IL-6	0.766	0.0360	0.721–0.806
CRP	0.768	0.0365	0.724–0.808
SOFA	0.845	0.0306	0.806–0.879
PSI	0.859	0.0310	0.821–0.891
Minor criteria	0.663	0.0421	0.615–0.709
	Difference	z statistic	*p* value
CIRP ~ PCT	0.0774	3.503	0.0005
CIRP ~ IL-6	0.107	4.883	< 0.0001
CIRP ~ CRP	0.105	4.683	< 0.0001
CIRP ~ PSI	0.0142	0.883	0.3774
CIRP ~ Minor criteria	0.210	5.892	< 0.0001
PCT ~ IL-6	0.0295	1.527	0.1268
PCT ~ CRP	0.0273	1.308	0.1908
PCT ~ PSI	0.0632	3.167	0.0015
PCT ~ Minor criteria	0.132	4.338	< 0.0001
IL-6 ~ CRP	0.00215	0.0961	0.9234
IL-6 ~ PSI	0.0926	4.994	< 0.0001
IL-6 ~ Minor criteria	0.103	3.427	0.0006
CRP ~ PSI	0.0905	4.818	< 0.0001
CRP ~ Minor criteria	0.105	3.883	0.0001
PSI ~ Minor criteria	0.196	6.483	< 0.0001

*AUC* The area under the receiver operating characteristic curve, *CI* Confidence interval, *CIRP* Cold-inducible RNA-binding protein, *PCT* Procalcitonin, *IL-6* Interleukin-6, *CRP* C-reactive protein, *PSI* Pneumonia severity index, *SOFA* Sequential organ failure assessment

## Discussion

The main findings of the current study comprise the following: Severe CAP patients meeting the major criteria had the highest concentrations of CIRP, PCT, IL-6 and CRP, SOFA and PSI scores, and mortality rates. The numbers of most predictive minor criteria present were strongly and positively associated with the concentrations of CIRP, PCT, IL-6 and CRP, SOFA and PSI scores, and mortality. The patients with non-severe CAP meeting the most predictive minor criteria demonstrated unexpectedly higher concentrations of CIRP, PCT, IL-6 and CRP, SOFA and PSI scores, and mortality rates, compared with the patients with severe CAP not fulfilling the criteria. CIRP was strongly linked to the major/minor criteria, SOFA and PSI scores, mortality, PCT, IL-6 and CRP, had a significant increased OR for mortality, and best predicted mortality, indicating that it might determine the severity of CAP and the presences of IDSA/ATS major/minor criteria and suggesting that it might be a novel and intriguing biomarker for pneumonia to monitor host response and predict mortality. A CIRP of ≥ 3.50 ng/mL might be more valuable cut-off value for severe CAP, with 61.4% sensitivity and 88.9% specificity.

Interestingly, the current study discovered that CIRP might determine the severity of CAP and the presences of IDSA/ATS major/minor criteria, had a significant increased OR for mortality, and best predicted mortality. The classic SOFA scoring system was well established and employed to assess organ dysfunction/failure [26]. The valuable PSI scoring system was created to evaluate pneumonia severity [4]. SOFA and PSI scoring systems are well-validated clinical tools and regarded as benchmarking. The IDSA/ATS major/minor criteria responsible for clinical triage were well validated [7–9]. The rank correlation coefficients between CIRP and SOFA and PSI scores were very high in the current study (0.843 and 0.952, respectively). As a result, CIRP might determine CAP severity, in other words, the presences of IDSA/ATS major/minor criteria, and ultimately the rates of mortality, which might have implications for more accurate clinical triage decisions. Future prospective multicenter larger cohort studies are warranted to assess the generalisability of the current findings.

An elevated PCT level was a risk factor for death from CAP (risk ratio 4.38, 95% CI 2.98–6.43), particularly in patients with a low CURB-65 score. For critically ill patients, an elevated PCT level was associated with an increased risk of mortality (risk ratio 4.18, 95% CI 3.19–5.48) [27]. But later studies favored the idea that PCT had limited predictive value. PCT is not an independent predictor of 30-day mortality, albeit predicts pneumonia severity in patients with pneumonia acquired outside the hospital [28]. PCT demonstrated moderate predictive value for the prognosis of hospitalized CAP [29]. Elevated IL-6 indicates a higher risk of 30-day mortality [84% sensitivity, 87% specificity and 0.934 AUC (95% CI 0.864–1.000)]. Moreover, IL-6 levels have been shown to have a good correlation with various clinical severity scores (e.g. PSI) [30]. Admission CRP <  100 mg/L has reduced risk for 30-day mortality, need for mechanical ventilation and/or inotropic support, and complicated pneumonia. CRP is an independent marker of severity in CAP [31]. In this study, CIRP was strongly associated with PCT, IL-6 and CRP, the correlations of CIRP with mortality and SOFA and PSI scores were the closest among these biomarkers, and CIRP was performed best in mortality prediction among these indices. Therefore, CIRP might be a novel and intriguing biomarker for pneumonia to monitor host response and predict mortality. Much more research is needed to investigate this matter, especially data on the trajectory of CIRP level during the patients’ hospital journey in particular correlation between witnessed decline in the value and patient length of hospital stays.

We found that the optimal cut-off value of CIRP level for predicting mortality was ≥ 3.50 ng/mL, which at least corresponded with the average value of severe CAP patients meeting two of the most predictive minor criteria, whose mortality increased to 16.7%, and that the mortality of patients fulfilling such high level rose sharply to 40.3%. Hence, this cut-off value might be regarded as a threshold to distinguish the patients with severe CAP who might require intensive care from those with non-severe CAP. This finding requires external validation before recommendation for decision making in clinical practice.

The well-validated IDSA/ATS major/minor criteria was widely used because it is a helpful tool to help stratify sick CAP patients, but the major problem associated with the minor criteria might be a lack of consideration of weight in prediction in clinical practice. We previously reported that the numbers of minor criteria present were not positively associated with SOFA and PSI scores and mortality [11], and that scored minor criteria orchestrated improvements in predicting mortality and severity in patients with CAP [32]. These might be envisaged to interpret the reason why the minor criteria were not strongly linked to SOFA and PSI scores and mortality, and predicted mortality worst in the current study.

### Limitations

Several limitations of this study deserve comment. First, the prospective cohort was derived from two centers in a city, but not multicenter settings located in different cities in different countries. This may limit the generalisability of the results. Second, 1637 patients were assessed with the result of 1611 patients eligible, but only 404 discontinuous patients were enrolled because of the limited financial support available from the foundations and the huge differences in the frequencies of major/minor criteria present, so the results should be viewed with caution. Had the number been larger, perhaps the results might have been more robust. FiO_2_ is not so accurate by mask flow. Finally, there were not enough severe CAP patients who fulfilled the major criteria or three of the most predictive minor criteria at the two departments, most of whom were admitted to general ICU.

## Conclusions

CIRP levels were strongly correlated with the IDSA/ATS major/minor criteria. CIRP might determine the severity of CAP and the presences of major/minor criteria and best predicted mortality, and a CIRP of ≥ 3.50 ng/mL might be more valuable cut-off value for severe CAP, suggesting that CIRP might be a novel and intriguing biomarker for pneumonia to monitor host response and predict mortality, which might have implications for more accurate clinical triage decisions.

## Data Availability

The datasets used and/or analysed during the current study are available from the corresponding author on reasonable request.

## Abbreviations

CAP: Community-acquired pneumonia
IDSA/ATS: The Infectious Disease Society of America and the American Thoracic Society
ICU: Intensive care unit
BUN: Blood urine nitrogen
WBC: White blood cell
PaO2/FiO2: Arterial oxygen pressure/fraction inspired oxygen
SOFA: Sequential organ failure assessment
PSI: Pneumonia severity index
CIRP: Cold-inducible RNA-binding protein
PCT: Procalcitonin
IL-6: Interleukin-6
CRP: C-reactive protein
CT: Computer tomography
SD: The mean ± standard deviation
ROC: The receiver operating characteristic
AUC: The area under the receiver operating characteristic curve
PPV: Positive predictive value
NPV: Negative predictive value
CI: Confidence interval
*r*_s_: Rank correlation coefficient. OR: Odds ratio
